# ‘No one size fits all’ – community trust-building as a strategy to reduce COVID-19-related health disparities

**DOI:** 10.1186/s12889-022-14936-6

**Published:** 2023-01-04

**Authors:** Margareta Rämgård, Rathi Ramji, Anders Kottorp, Katarina Sjögren Forss

**Affiliations:** Department of Care Science, Faculty of Health and Society, Malmö University, Jan Waldenströms gata 25, SE-20506 Malmö, Sweden

**Keywords:** Health promotion, Culture brokers, Pandemic, Resilience, Vulnerable neighbourhood

## Abstract

**Background:**

Citizens with low levels of social capital and social status, and relative poverty, seem to have been disproportionally exposed to COVID-19 and are at greater risk of experiencing poor health. Notably, the incidence of COVID-19 was nearly three times higher among citizens living in socially vulnerable areas. Experiences from the African Ebola epidemic show that in an environment based on trust, community partners can help to improve understanding of disease control without compromising safety. Such an approach is often driven by the civil society and local lay health promoters. However, little is known about the role of lay health promoters during a pandemic with communicable diseases in the European Union. This study had its point of departure in an already established Community Based Participatory Research health promotion programme in a socially disadvantaged area in southern Sweden. The aim of this study was to explore how citizens and local lay health promoters living in vulnerable neighbourhoods responded to the COVID-19 pandemic a year from the start of the pandemic.

**Method:**

In-depth interviews with the 5 lay health promoters and focus group discussions with 34 citizens from the neighbourhood who were involved in the activities within the programme were conducted in autumn 2020. The interviews and focus group discussions were transcribed verbatim and analysed using qualitative content analysis following an inductive approach.

**Results:**

Four themes emerged including, ‘balancing between different kinds of information’, ‘balancing between place-based activities and activities on social media’, ‘bridging between local authorities and the communities and community members’, and ‘balancing ambivalence through participatory dialogues’.

**Conclusion:**

The study highlights that a Community Based Participatory Research programme with lay health promoters as community trust builders had a potential to work with communicable diseases during the pandemic. The lay health promoters played a key role in promoting health during the pandemic by deepening the knowledge and understanding of the role that marginalised citizens have in building resilience and sustainability in their community in preparation for future crises. Public health authorities need to take the local context into consideration within their pandemic strategies to reach out to vulnerable groups.

## Background

Despite the global lockdown and strict restrictions owing to the COVID-19 pandemic, Sweden chose a different strategy to minimise the spread of the virus compared to other countries, in that total lockdowns were not implemented. Above all, the government focused on alleviating public concerns and demanded the population to take collective actions by implementing effective and timely measures and useful information from the public health authorities (PHA) [1]. The efforts of the PHA focused on disseminating information through many different channels such as the media, local and state authorities, as well as parts of civil society organisations such as the Red Cross, while the health services mainly remained focused on healthcare provision. However, a major challenge for collective action was that there was a distance between the PHA at the national and regional level, who defined the problem, and marginalised communities at the local level who were supposed to benefit the most from these initiatives but seldom influenced the decision-making process [2, 3]. Yet another problem in applying this collective action strategy was the population heterogeneity in terms of culture and even the understanding of the problem owing to individual, emotional and cognitive challenges [3].

### COVID−19 in vulnerable communities

The overall public health strategy in Sweden was framed without taking into consideration the vulnerable communities living in socially deprived areas. The focus was mainly on the- population at risk for non-communicable diseases and elderly over 65 years [4]. According to Bambra and colleagues, the pandemic occurred against the backdrop of a rising rate of non-communicable diseases (NCDs) and unequal exposure to the social determinants of health, making it a more syndemic pandemic, which has further exacerbated the existing NCDs and social conditions [5]. Further, it is argued that in Europe, and in the US and the UK, the poorest parts of the population, who were also predominantly ethnic minorities and migrants, experience more chronic diseases in general, and were therefore also more likely to be affected by the COVID-19 [6]. Consequently, it is regarded as important to be aware of the circumstances revolving around the COVID-19 pandemic also from an equity perspective, paying attention to vulnerable communities and initiating actions in these communities. Marmot et al. has also highlighted that the gap in health equity has increased in socially deprived neighbourhoods during the pandemic [7].

In Sweden, nearly 32% of the population affected by COVID-19 were citizens living in less affluent neighbourhoods in bigger cities, where 5.4% of the country’s population reside [8]. These neighbourhoods are frequently occupied by migrant and minority groups who differ from the general population with regard to their immediate physical environment, housing conditions (crowded living, several generations living together), education levels, occupational status and even access to the job market [4]. Besides ethnic, cultural and class-related aspects that remain largely ununderstood, there have also been barriers for the authorities to gain information about the citizens living in these areas and thus they are often underrepresented in the population registers [9]. Furthermore, the trust in authorities is low among these populations owing to their experiences from their home countries and to language barriers that restrict their access to information provided by the local authorities [9]. Prior research shows that during the pandemic many of those residing in these neighbourhoods followed news and recommendations from their own homelands through the internet and social media and then experienced a sense of confusion given the differences in the recommendations [10, 11]. Some of them were not aware of the Swedish recommendations and tried to follow the advice of their family, friends and authorities from their homelands since the PHA had not addressed these uncertainties through local actions. Thus, the communities did not understand why Sweden chose a different path of pandemic control [12].

Community health promotion can be the need of the hour to successfully address the challenges posed by the COVID-19 pandemic through providing support to handle the social and health effects of the pandemic [13]. An important part of health-promoting work is to reach out to this population and disseminate information that caters to this group. Previous experiences from similar emergency situations show that communication which is also an important part of health promotion particularly during a crisis should be adapted to the target audience and their local situations [14, 15]. For example, to meet people in their own environment in order to communicate, adapt, and help interpret information to them. Thus, it is important for authorities to initiate work through channels that are trusted among the population [3, 16, 17]. Based on experiences from the Ebola epidemic, the use of local brokers who are members of the same neighbourhoods in different community-based programmes, together with academia and other stakeholders, has been fruitful to ensure trustworthy relationships in the communities [18].

### Brokers in the community

A similar broker initiative within a community-based participatory research (CBPR) programme for promoting health, Equal Health*,* was established in 2016 and implemented in Malmö in southern Sweden in 2017. The overall aim of the programme was to reduce health inequalities in socially deprived areas in Sweden through CBPR and community health promotion [19, 20]. Community-based participatory research (CBPR) is an approach where academia and different stakeholders can work collaboratively with the communities during pandemic situations to establish local initiatives for communication through collective action [21, 22]. Further, CBPR focuses on integrating the community in decision-making processes and encouraging them to actively participate in the research process so as to create a common understanding of the local problems and practices specific to their own context. Such an effort is said to result in the identification of innovative strategies to promote social changes [23]. The programme implemented in Malmö had local brokers from the area, called lay health promoters (LHP), who facilitated different health promotion activities within the programme. The programme’s health promotion activities were based upon the citizens’ needs in the area and the LHPs were therefore in constant dialogue with the citizens in the community about their needs, and had support of local stakeholders from social care, health care and non-governmental organisations for organising the health promotion activities [24]. Before the COVID-19 pandemic the LHPs in the programme had built up a community capacity that was very useful for local collective actions during the pandemic. This community capacity, where LHPs have a key role, is also the context for the current study.

The LHPs who were involved in the CBPR programme also continued to work towards assisting the communities together with local stakeholders during the COVID-19 pandemic. The CBPR programme in Malmö was built upon Paulo Freire’s concept of empowerment [25] and the LHPs were educated to participate in the programme with a goal to empower citizens in the community.

According to Bhaumik and colleagues, community health workers (CHWs) played a critical role in the pandemic [26]. However, they also highlight the need to ensure role clarity, so that the CHWs can work efficiently for the benefit of their own community. In order to ensure long-term commitment and sustainability, health communication must be collaborative, respectful, inclusive and above all directed by the community [27]. Research on a CBPR programme among indigenous populations in the US has drawn attention to the importance of considering cultural aspects to achieve a more holistic COVID-19 response. Further, a need for culturally relevant psychosocial support during the COVID-19 pandemic also emerged from the same context [27, 28]. Cultural brokers play an important role as bridges between the society and the citizens.

As the Swedish national strategy was built on taking personal responsibility in the collective action during the COVID-19 pandemic, there was a need for increasing local access to trust-worthy information and support to understand such information. Such an initiative should also focus on preserving the health and well-being of populations living in vulnerable neighbourhoods. Research has shown that approaches driven by civil society and LHPs are important to prevent both non-communicable and communicable diseases. However, studies on the role of LHPs in preventing infectious diseases in a European, as well as the Swedish context, are scarce.

Thus, the aim of this study was to explore how citizens and local LHPs’ living in vulnerable neighbourhoods responded to the COVID-19 a year from the start of the pandemic.

## Methods

### Study design

A qualitative, descriptive design was adopted for this study as it is used for explaining and describing experiences using participants' own language [29]. A more detailed description of the complete design and structure of the health promotion programme can be found in a previous study by the research team [20].

### Sample

Five of the LHPs who were involved in the larger program who also lived in the neighbourhood were included in this study. The LHPs were all females aged between 33–56 years. They were all first-generation immigrants of different age and ethnic background and represented the countries of origin of the population that was predominant in the neighbourhood (Iraq, Syria.

Lebanon, Palestine, Iran och Sudan). Four of the LHPs were from Arabic speaking countries. One of the LHPs who worked with older adults with mental illnesses and functional disabilities was from Eastern Europe. Further to gain a wider perspective regarding the LHPs work during the pandemic 34 women from the community aged between 26–78 years were included in this study. They came from Arabic speaking countries including Iraq, Syria, Lebanon, Palestine, Iran and Sudan. These women were those who were involved within a part of the health promotional program focusing on physical activity facilitated by the LHPs. They also were in contact with the LHPs throughout the COVID-19 pandemic. A demographic characteristics of the LHPs and the women from the neighbourhood is presented in Table 1.

**Table 1:** Characteristics of participants in the study

Participants	LHP	Citizens
	**(*N* = 5)**	**(*N* = 28)**
***Gender***	Female (5)	Female (28)
***Age Group***	33—56 years	26 -78 years
***Employment status***		
*Employed*	5	2
*Sick leave*	**-**	3
*Parental leave*	**-**	1
*Studying/Internship*	**-**	8
*Retired*	**-**	7
*Unemployed*	**-**	7
***Educational qualification***		
*University education*	1	7
*High school*	2	10
*Elementary school*	2	11
***Land of origin***		
*Iraq*	2	10
*Syria*	-	6
*Lebanon*	1	4
*Palestine*	-	4
*Iran*	1	3
*Sudan*	-	1
*Hungary*	1	0

### Setting

The study took place in a geographical area of Malmö (the third biggest city in Sweden) with a high rate of unemployment and crime, low education levels and also a significant prevalence of health inequalities. The area has been considered one of the twelve most socially deprived areas in Sweden [24, 30]. A majority of the residents are first-and second-generation migrants [24]. Studies accounting for COVID-19 in the district in which this area is a part shows a high rate of infection among the inhabitants. Further it has been reported that the number of working days lost due to illness per individual in this neighbourhood aged between 20–64 years during the year 2020 was 50% higher than the average rate in Malmö city.

### Sampling and recruitment

The members of the research team were also part of the steering group within the larger program. This group was a dialogue forum for the LHPs and other societal actors before and most importantly during the pandemic. The research team contacted the LHPs during the local steering group meeting and invited them to participate in this study. In parallel to participating in the in-depth interviews the LHPs also facilitated the discussions with the women in the neighbourhood in the physical activity group. The LHPs used a WhatsApp video call in Arabic to inform the women and invite them to the focus groups. All invited participants agreed to participate in this study.

### Data collection

#### Individual interviews with LHPs

All five LHPs who were engaged in the CBPR programme were interviewed during year 2020. Initially, a focus group interview was planned. However, owing to the COVID-19 pandemic restrictions it was not possible to meet in person, and consequently individual interviews were conducted via Zoom by the first author. The interviews aimed to get an understanding of how the LHPs had experienced the participants’ knowledge about the pandemic, the participants’ health behaviours during the pandemic (if and how they had changed), and how citizens responded to the communication of information from authorities and media during the pandemic. When necessary, probing questions, e.g., ‘Can you please give an example’ or ‘Can you tell me more, please’, were used. The interviews were performed by the authors in Swedish and proceeded until no new information was identified. They lasted on average between 35—50 min and were digitally recorded.

#### Focus group interviews with citizens from the neighbourhood

Focus group interviews were held with the women from the neighbourhood during autumn 2020. The interviews were held when there was a temporary lift in the COVID-19 recommendations against gathering in public spaces in early autumn 2020. Three LHPs working within the CBPR programme, together with the research team, were involved in contacting the women and facilitating the data collection. The research team ensured that participants had no flu symptoms or other general health ailments that could hinder their participation. A total of four focus group discussions were conducted with six to eight women in each group was facilitated by the second author, while the first or the fourth author acted as observators alternatively. A detailed description of the composition of each focus group is presented in Table 2. Large spacious rooms in the social meeting place owned by the municipality where activities within the larger program happen. The seating arrangements were made in a way that it was possible to maintain a two-meter distance, between participants during the discussions. Participants were provided with detailed information about the purpose of the study by the research team. Further, they were informed that the discussions would be audiotaped and ensured that the material would be used only for research purposes. Each focus group interview lasted between 1 and 2 h and discussions proceeded until no new information was identified. The research team engaged in the discussions primarily in Swedish, with the LHPs translating back and forth since the participants mainly spoke Arabic. The subjects discussed during the focus group interviews aimed at understanding participants’ experiences during COVID-19 and the potential benefits of having participated in the CBPR programme [19]. The interviews were based upon a thematic CBPR guide by Wallerstein [31] designed to evaluate processes related to empowerment and participation in the collaboration. The guide had been used as a process evaluation tool during the CBPR programme, as a continuous evaluation of the programme together with the citizens to evaluate and adjust activities.

**Table 2:** Composition of the focus group discussion with the participants

	Citizens participating in the four focus groups			
Demographic Factors	Group 1	Group 2	Group 3	Group 4
	*N* = 7	*N* = 7	*N* = 8	*N* = 6
*Age group*	35–69 yrs	33 – 65 yrs	33- 78 yrs	23–53 yrs
*Employment status*				
*Employed*	2	0	0	0
*Sick leave*	0	2	1	1
*Parental leave*	0	0	0	1
*Studying/Internship*	0	4	2	2
*Retired*	2	1	4	0
*Unemployed*	3	0	1	3
*Educational Qualification*				
*University education*	2	0	3	2
*High school*	3	3	1	3
*Elementary school*	2	4	4	1
*Land of origin*				
*Iraq*	4	1	2	2
*Syria*	1	1	3	3
*Lebanon*	1	2	1	1
*Palestine*	0	1	1	1
*Iran*	1	1	1	1
*Sudan*	0	1	0	0

The CBPR guide is focused on dimensions of experiences of power and trust in the community and local partners, the degree and experiences of participation in the activities, the skills acquired and the outcome of the health promotion activities. During the pandemic, the dialogues about the outcome also focused on the pandemic situation in the community. The participants were requested to elaborate on aspects about how their lifestyle, health routines and living conditions had changed during the COVID-19 pandemic, what they had learnt about health promotion activities related to the pandemic as participants in the CBPR programme, and what information about lifestyle changes related to the pandemic they had received.

### Data analysis

All interviews were transcribed verbatim and then analysed using an inductive qualitative content analysis as described by Elo and Kyngäs [32]. The transcripts were read several times by all the authors to gain an overall understanding and also to reduce the risk of prejudice [33]. Thereafter, meaning units that corresponded with the aim of the study were condensed and labelled with a code, derived from the data manually. The codes were interpreted and compared for similarities as well as for differences regarding phrases and patterns without losing their content. Finally, the codes were transformed into tentative themes. The analysis process is presented in Table 3.

**Table 3:** Examples of the process of analysis

Meaning units	Condensation	Codes	Subthemes	Themes
‘When the regular group activity was cancelled, I started using the stairs here every day instead of the elevator. And I washed and did the dishes and even did gardening in my balcony. It’s the same as exercise because I’m not just sitting but moving my body. Because I have learnt that physical activity is important’(Woman from the neighbourhood) ‘I think the successes in the work have been the digital contacts, everything from WhatsApp groups to Facebook, and other things, these systems are so established, we already used it in our work for sharing health tips, recipes, and advice with each other. All these groups existed even before the pandemic, so it was much easier to work during the crisis when you could meet, since it was already established in the programme’ (LHP)	Group activities started before the pandemic Activities based at home during the pandemic Advice and activities through social media before and during the pandemic Health promotion advice to citizens through already established channels using social media	Group activities started before the pandemic Activities based at home during the pandemic Advice and activities through social media during the pandemic Health promotion advice to citizens through already established channels using social media	Facilitating health promotion activities before and during the pandemic Adapting health promotion activities based on the needs of the communities	Balancing between place-based activities and social media

### Trustworthiness

The current study was conducted in accordance with the criterias for trustworthiness as recommended by Guba and Lincoln such as credibility, transferability, dependability, and confirmability [34]. This study was part of a larger health promotional program which is built on principles of trust and mutual respect. Thus, the pre-established contact based on trust between the researcher and the participants facilitated understanding and interpretation of the data collected. The participants also had the opportunity to share trustworthy accounts ensuring credibility. We strived for transferability by sampling participants from diverse backgrounds and age groups and also through including different perspectives from a multicultural disadvantaged context. Further to get a deeper understanding of the subject under study, multiple perspectives were included and consequently, data consisted of two different methods. The two groups of participants were interviewed separately so one group does not take precedence over the other. The data coded by the first two authors following preliminary analysis was reviewed by the last two authors who were not involved in the data collection thus achieving dependability. The last two also rechecked audio recordings in iterations to ensure confirmability. Further, member checking was done at the end of all interviews and focus groups through briefly summarising the discussions to the participants. Further the transcripts and the final results were presented to the participants to reconfirm the interpretations captured by the research team.

## Results

The LHPs’ experiences of working with the citizens in the neighbourhood during the pandemic have been described using four themes, namely, ‘Balancing between different kinds of information’, ‘Balancing between place-based activities and activities on social media’, ‘Bridging between local authorities and the communities and community members’, and ‘Balancing ambivalence through participatory dialogues’. The code layout is presented in Table 4.

**Table 4:** Coding layout

Codes	Subthemes	Themes
Concerns regarding children’s well-being Influence of information from home countries Rumors regarding COVID-19 in the community Social media as platform for exchange of information Mistrust in written information from Public Health Authorities	Mistrust in information about COVID-19 Health promoters as trustworthy sources for information	Balancing between different kind of information
Group activities started before the pandemic Activities based at home during the pandemic Advice and activities through social media before and during the pandemic Health promotion advice to citizens through already established channels using social media	Facilitating health promotion activities before and during the pandemic Adapting health promotion activities based on the needs of the communities	Balancing between place-based activities and social media
Authorities lack understanding Language barriers Difficulties to making appointments to meet the doctor LHPs help establish contact with health and social care	Authorities’ lack of understanding for sociocultural factors that influence communication LHPs role in providing technical and communication support to reach health and social care	Bridging between local authorities and the communities
Confusion following listening to different opinions Discussion within the health promotion activity group from the programme Change of opinions after reflecting with the group Spreading knowledge gained to friends and family in other countries	Engaging in group dialogues LHPs as facilitators of reflective dialogues	Bridging ambivalence by reflective dialogues

### Balancing between different kinds of information

At the start of the pandemic, one of the LHPs stated that several kinds of information spread rapidly in the neighbourhood because it was obtained from different sources, including social media. An important experience that the LHPs gained from working closely with the citizens was the need to deal with the situation where citizens got too much information from different sources and became confused. One common piece of misinformation that spread was that the pandemic was caused by destructive capital forces aiming to take over the financial market and thus many citizens were not careful and did not follow the recommendations.

‘Well, that’s the double signals you get when someone comes up with a rumor that the COVID-19 pandemic was the start of some financial crisis. Some citizens were like … simply didn’t believe it, while others were terrified anyway.’ (LHP, age 49 and elementary school educated)

Yet another piece of information which spread among the citizens was that ethnic minorities were more exposed to the virus because of their ethnicity. Sources on the internet also projected the pandemic as a ‘foreign’ problem and thus some citizens in the neighbourhood were negligent and did not care about following recommendations and precautions to prevent them from being exposed. Further, they seemed to be concerned about how these circumstances affected children’s health.

‘After all, children experience the world both from their parents’ concerns and what they see themselves, so I think there have been a lot of reflections on what is happening abroad, and what is happening here..., that is, there have been strange questions asked …., like if you come from a certain country or ‘colour’ it’s easier to get sick and that it’s due to genetics and this kind of thing, suggestions that the Corona virus was more connected to different ethnic groups.’ (LHP, age 38, and high school educated)

It also emerged that receiving mixed information confused the citizens and further triggered pandemic fear among the families. Moreover, as the Swedish schools remained open during the pandemic, unlike other parts of the world, many parents were reluctant to send their children to school although the state recommended that families do so. However, one of the LHPs stated that even those parents that trusted the PHA and believed in their analyses and recommendations during the pandemic, kept the children at home, fearing the risk of exposing them to the virus since many children used public transport to commute to schools situated in other parts of the city. The LHPs communicated the PHA’s recommendations regarding why the schools had to be kept open since the children’s psychological well-being was influenced positively by maintaining belonging to their social context in the schools. Further, the LHPs explained to the parents that the children might need additional support from their parents when offered distance education, that all children might not have access to the necessary support, and that the learning process might not be effective when done via the internet. The LHPs also had to clarify that schools were not arenas where the pandemic spread rapidly.

‘I heard that in other countries the virus affects even the children, so the schools were closed. Therefore, I was afraid to send my children to school. But three weeks after having them home the school announced that we had to send the children to school, but I was very unsure. Then the health promoter explained to me about this, and I sent the children to school again.’ (Women from the neighbourhood, Age 48 , and university educated)

The LHPs stated that the citizens preferred to read Swedish texts directly from the web sources of the PHA and the healthcare authorities instead of translating them into their own language, as they did not trust the translations.

‘So, people (if I am honest) have very low trust in other authorities, but they have a lot of trust in us. I need to read the Public Health Agency information in Swedish, then I send [it] to the group through a WhatsApp (saying they translate the information correctly).’ (LHP, age 34 and high school educated)

The LHPs acted as translators, and explained the information to citizens, which led people to embrace the information and comply with the restrictions.

### Balancing between place-based activities and activities on social media

At the beginning of the pandemic, outdoor activities were initiated as part of the health-promoting programme in place of the regular activities, and they were coordinated by the LHPs where citizens gathered in small groups for yoga and light gymnastics. Citizens believed that, besides promoting health, these activities also helped reduce the perceived anxiety caused by the pandemic.

‘At the beginning of the pandemic, there were no regular activities, and we were very sad. Later, the health promoters sent us information about activities like yoga and gymnastics or even other things on the WhatsApp group. We had activity three times a week, but all outside in the yard or in the park. We could not be all be there at the same time since they split us up into smaller groups, but it was still fun.’ (Woman from the neighbourhood, age 48 and elementary school educated)

During late autumn 2020, when the spread of the virus increased rapidly throughout Sweden, the LHPs decided to instead coordinate the activities through social media, so that the groups could continue engaging in physical activity, yoga and dance from home despite the pandemic. Engagement in these online activities helped reduce feelings of loneliness and isolation while also promoting health. Moreover, the citizens made a conscious effort to be physically active in their everyday life within their homes by, for example, using the staircase instead of the elevator in their buildings.

‘When the regular group activity was cancelled, I started using the stairs here every day instead of the elevator. And I washed and did the dishes and even did gardening in my balcony. It’s the same as exercise because I’m not just sitting but moving my body. Because I’ve learnt that physical activity is important.’ (Woman from the neighbourhood, age 54 and high school educated).

To increase well-being in the community, health promotion information packages were sent out to households by the LHPs in collaboration with non-governmental organisations. These packages included information about COVID-19 together with presents for the children and LHPs’ contact information. The LHPs also initiated dialogues via social media, which created opportunities to continue with the activities even when restrictions to meet in public meeting places were introduced in an attempt to reduce the spread of the virus. Having moved to a digital forum seemed to have also attracted more participants to the group than before the pandemic.

Although these online activities worked well, the women and children concluded that they still longed for the physical meetings that energised them and made them perceive a stronger sense of community. Many of the women were low on finances and lived in small, cramped apartments. Several of them also lacked confidence regarding the advice they could get from each other when they were not meeting face to face. Furthermore, the physical meeting places were important locations since they were open for all citizens in the neighbourhood, including the women. The women said that through establishing social contacts with others in these physical places strengthened their sense of being in control of their life situation.

‘Working out yourself at home is boring. Meeting people helps me relax. It’s enough to see that people smile, talk and share our thoughts with each other in order to feel good even if you are yourself in the worst situation.’ (Woman from the neighbourhood, age 29 and high school educated)

According to the LHPs, it was difficult to initiate social processes between different groups via the internet, and all citizens did not have access to the internet even if they tried to help each other. However, the LHPs found it easier to support groups who were previously engaged in the health-promoting activities during the pandemic since they had worked with social media and WhatsApp to support the groups between the regular meetings. They also provided general social support and community information about the Swedish society and helped them in many ways over the internet and via cell phones. The experience of using social media for coordinating the health-promotional activities proved very effective during the pandemic, as the LHPs were able to continue with health promotion activities, combined with dialogues regarding the instructions from the PHA, via the WhatsApp groups. Using these digital contacts, they could also quickly respond to the citizens’ queries about the state regulations.

‘I think the successes in the work have been the digital contacts, everything from WhatsApp groups to Facebook and other things. These systems are so established, we already used it in our work for sharing health tips, recipes, and advice with each other. All these groups existed even before the pandemic, so it was much easier to work during the crisis when you could meet, since it was already established in the programme.’ (LHP, age 36 and high school educated)

By participating in the activities, the LHPs were able to promote the health of families through various types of physical activities and also help them maintain a good diet, but the most important thing, according to the citizens in the area, was the opportunity to remain together and create hope for the future. This also reduced their concerns about becoming infected and made them think about something else than the ongoing pandemic.

‘Lack of knowledge on how to reach groups, language barriers, isolation, technical obstacles that prevent information from coming out to the communities. The health promoters remove all these knots that everyone else (in the society) has experienced and but that have never been resolved.’ (LHP, age 56 and university educated)

### Bridging between local authorities and the communities

Several migrant groups in the neighbourhood did not know how to use the support they received from primary care and neither did they understand the advice they got on telephone from the healthcare services. On the other hand, there was no formal relationship between the LHPs and the healthcare system either. Some families especially the newcomers and refugees turned to the LHPs because of language barriers. On several occasions, LHPs even had to acutely call for an ambulance since some citizens remained at home too long with a severe shortness of breath and did not know whom to contact.

‘We help people book appointments, for example, in primary care. Some could not call 1177 since they do not respond to them because they have a very difficult language (dialects).’ (LHP, age 49 and elementary school educated)

But it was not only the language that was the problem for citizens with the primary care.

‘When it comes to health care, language is not the real problem, we can get someone to translate, but we don’t get the support we’re looking for, they don’t understand what we need.’(Women from the neighbourhood, age 65 and elementary school educated)

The LHPs also provided additional support to poor families, new arrivals and citizens that needed help to get access to social care. The LHPs conveyed, and supported them in filling out, forms for authorities and were a general social support. Each of the LHPs was in contact with over 60 families.

‘Some of them are very poor ... between 15 and 20 families who have a very difficult situation (in my area), so I contact them every day and they contact me sometimes and they want advice and tips about what to do about their situation.’ (LHP, age 34 and high school educated)

Citizens with cognitive disabilities that were living at home were the most isolated group because they belonged to a pre-defined risk group. They could not always understand the information provided by the PHA and the health and social care sector. Because of their disabilities they were also not all able to use the internet independently, so for these citizens the contact with the LHPs was the only way to get actual help and support as well as reducing loneliness and isolation. The LHP created a group phone call to ensure that citizens with cognitive disabilities could manage their lives and get the right information from the PHA.

Although they were not physically present in the neighbourhood, social service field workers were committed to work from a distance and were in contact with the LHPs. They could receive information through the LHPs about older citizens, risk groups and the general well-being of the community and also delivered important information to the community via the LHPs.

‘Sometimes I also find it difficult to come up with a solution, but when I talk to field social worker ... I come up with idea and we find a solution together ... For example, we were asked very strange questions regarding corona. I have brought them to the field workers from social care and we have found answers and passed on to the group.’ (LHP, age 56 and university educated)

### Bridging ambivalence by reflective dialogues

The LHPs picked up on these concerns and worked with reflective dialogues, in meetings both outdoors and through social media, especially on WhatsApp. During the COVID-19 pandemic, they used this knowledge to work with citizens’ ambivalence, balancing different opinions.

‘Some are careless in keeping their distance and washing their hands and they do not believe that there is a COVID-19 disease. Some others, they’re very worried and they’ve just isolated themselves. And we have to be in the middle balancing between them all.’ (LHP, age 38, and high school educated)

One such example was seen in the group of older migrants. Some of them obtained news about COVID-19 from newspapers in their home countries and compared this with news from media in Sweden. They were not sure which news they could rely on. The group of citizens and LHPs could then engage in continuous reflective participatory dialogues, given that they trusted each other even before the pandemic. During such discussions the group of citizens and the LHPs seemed to have found their own rationale for how to deal with different messages about COVID-19 from social media, home countries and authorities.

‘He read in the Romanian newspapers that COVID-19 it is just a humbug, it’s not true, it’s only the West that wants to sell medicines because medicine sales are low, and he took it very seriously that it has to be so and so he tried to persuade several others too. But after the discussion in my group, most people have said that “No, we trust the LHP”, so I feel proud, that trust is intact.’ (LHP, age 56 and university educated)

On the other hand, the citizens also stated that the messages from the PHA in Sweden differed from the COVID-19 information provided in their home countries. They started to reflect on the fact that different countries worked differently and that this had to be based on different kinds of strategies that were, in turn, based on the local context. They also came to understand that neither of the strategies is wrong but that they may work only in the respective context. After reflecting together with the LHPs, they concluded that they had to follow recommendations from the country they lived in for the moment.

‘At the beginning I got the news from my country, and in Sweden. But the rules here in Sweden were different. There’s a big difference. I later understood, after discussing with the LHP, that I have to choose to follow the rules of the country where you live. And for my parents and other family members who live in our homeland, it differs because it’s another country. We listen to the news from there also just to know how they are doing. If anything happens there. But it’s their rules, their situation in that country, not here. I can’t do anything for them. I can only listen to the news from my country.’ (Woman in the neighbourhood, age 35 and elementary school educated)

The LHPs not only spread the relevant information in the immediate neighbourhood but also spent much of their time spreading the PHA recommendations to their neighbours and to friends in other countries as well. They even invited their friends and relatives to the yoga sessions and aerobics via social media and kept them updated with information about why Sweden was open during the restrictions.

‘I find in YouTube, WhatsApp, the internet that information. I send to my relatives and friends, always, if anything is good, I send. I share with people. I put on Facebook too, because all friends watch it. Yes, I spread everything I read. I’ll send to my brother who is sick and has Corona. All my family is in Syria, and I send it to them.’ (Woman in the neighbourhood, age 54, elementary school educated)

## Discussion

The results of this study show that the citizens felt that the LHPs played a crucial role in supporting the community to counteract problems related to both communicable and non-communicable diseases, through addressing concerns with information and needs to withstand the pandemic stress, while also helping to maintain their health and lifestyle. The citizens reflected with the LHPs working with their ambivalence about different sources of information, the lack of support from the health care and social authorities and the lack of health promotional activities. The reflective dialogues and the health promotion activities adapted to the COVID-19 situation facilitated by the LHPs helped the citizens reduce their worries and confusion while also help maintain their health during the pandemic. This shows that the civil society can be effective in supporting each other during the pandemic.

The LHPs had built up trust among the citizens long before the pandemic started, in the Equal Health programme. During the pandemic, the citizens felt that the LHPs had an important role in balancing and bridging the mixed information about COVID-19, at the same time as they facilitated situationally responsive health-promotional activities during the pandemic. Although few CBPR programmes started during the pandemic, research has confirmed that the most successful programmes were reported to be those that were initiated and organised based on trust-building processes through reflective dialogues and activities with the communities before the pandemic [35–37].

The results of this study show that the LHPs had an important role in balancing between different kinds of information that the communities received from both national and international sources, especially via social media, which created an uncertainty not only among adults but also among children. Prior research [38] also suggests that considering how information is communicated is only one side of the coin, since how the delivered information is interpreted is what determines change in action and behaviour during crises. Thus, it must be noted that the needs and perceived abilities of communities must be taken into account when delivering information, since ‘One size does not fit all’ [17]. In addition, Smith and Judd argue, when health-related information is not discussed but only distributed by the healthcare sector and other sectors, particularly during acute situations as the pandemic, it is critical to organise it in a concise and meaningful way ensuring that only necessary information reaches the citizens, as too much information, even if well intended, may create confusion [39]. However, it must also be noted that trust built through dialogues is a critical element in ensuring effective communication and initiating collective action, and the lack of trust in the authorities is an ongoing problem in similar populations. Several citizens in the neighbourhood had problems getting access to support from health and social care during the pandemic because of language barriers and lack of time to engage in dialogue with the citizens. The LHPs became an important bridge to the local authorities and health care through helping citizens understand the communications received from these authorities. This is in line with previous research arguing that LHPs could act as bridges for communication while also building trust between the healthcare sector, the social services and marginalised groups [40]. Besides providing support through information, the LHPs were also balancing between place-based activities and activities on social media during the pandemic. Promoting health through social media, coordinating outdoor activities and facilitating participatory reflective dialogues between the citizens during the pandemic was important because it helped the citizens both by reducing anxiety over the pandemic and promoting their health. This worked out well since the citizens had earlier experiences before the pandemic of social media group activities in the CBPR programme. In line with the current study, a previous study also identified the vital role played by LHPs in building social networks within smaller groups, which helped form a bridge with other members of the community and which may have led to building community capacity [41]. Community capacity can facilitate resilience during acute situations like that of the COVID-19 pandemic [42]. Community resilience is the ability of a community to take advantage of existing resources, internal as well as from their immediate external environment, with an overall aim to improve health and well-being while also sustaining health and recovery during crisis situations [ibid]. The LHPs were persons living in the community who initiated activities and participatory dialogues, which stimulated citizens to get better control of their life despite receiving contradictory messages from different sources.

The concept of brokers related to non-communicable diseases should be highlighted more in the European healthcare system, and the findings from this study contribute with important knowledge and understanding of how this role can support marginalised citizens during a crisis such the pandemic. Also, LHPs build trust in authorities, addressing epistemic injustice, and they can therefore act as a bridge over to the healthcare sector and other stakeholders ahead of a crisis. Epistemic injustice arises where power differences become exacerbated, particularly when this involves a certain individual or a community being regarded as less credible compared to other societal actors who are seen as providers to them [43]. Epidemic injustices appear when actors in the society (such as the PHA) do not see citizens as equally credible as professionals with regard to providing information about their situations during the pandemic. This power can unconsciously be misused when citizens do not speak the local language nor have the same norms and values as the society at large.

Schooled in the spirit of Freire in the CBPR programme [44], the LHPs listened to what the citizens said, and initiated dialogues, built on trust, where they worked on the ambivalence the citizens perceived on receiving different messages from social media and various authorities. CBPR involves reflection and dialogues as a method and has an emancipatory stance which is well-aligned with the ambitions of community empowerment highlighted in WHO declarations and reports on health promotion [45]. This emphasises that the LHPs work with and for communities, not only to communicate centralised ‘expert’ knowledge and recommendations informs that can be understood or applied locally, but also to strengthen communities’ capacity and address social determinants of health.

In line with this, Freire [46] also discusses the concept of the banking model of education that denotes authoritative hierarchy, where care providers and other societal actors see themselves with more power and position in the society than those in disadvantaged situations. The actors are more interested in providing knowledge to the communities rather than considering tapping on the communities’ own strengths [46]. This attitude is reflected in the discourse around health promotion and the role of the LHPs in the context where this study was constructed, a discourse aiming at ‘informing residents’ with an immigrant background about how the Swedish system works. The present study shows that the less fortunate can, through local support from the LHPs, rather than the authorities, themselves help revive and empower communities. The critical aspect of collective actions (such as the pandemic strategy in Sweden) is that the communities have access to resources that they can use to their advantage. The findings from this study show that the LHPs have been such a resource to the communities, helping them both promote health and prevent communicable and non-communicable disease. The LHPs seemed to have not just delivered information but also dealt with citizens’ ambivalences through reflective dialogues. During these dialogues the communities could themselves reflect on and reason out their concerns together, thus creating their own understanding of the situation without merely being forced to follow recommendations that they did not understand. Dialogue is a participatory reflective interaction process between people, while provision of information is a one-sided communication [37]. A reflective participatory dialogue results in deep learning where the learning process takes individual differences in the group into account and is frequently based within the everyday life of the learner [47].

Our study suggests that health promotion programmes built upon CBPR, and the theories of Freire, could build resilience for future crises. Freire’s pedagogy is based on a fundamental trust in people, in their knowledge, and in their capacity to grow and develop [48]. Here it is not just a question of inhabitants in the community coming to trust outside actors involved in this practice, but to what extent the various external stakeholders in the health-promotional programme actually trust the ability, knowledge, and expertise of the inhabitants in defining the change they want to achieve. The LHPs tried to overcome the issue of epidemic injustice and were trained in collaborative dialogues before the pandemic emerged.

### Strengths and limitations

The findings from the current study should be considered in the context of its limitations. The study was based on a relatively small and homogeneous sample, which could have implications regarding the transferability of the results. All participants were women, as one of the approaches of the CBPR programme was to focus on women with poor knowledge of Swedish in the area where the study took place. A qualitative design was found to be the most appropriate, since it is in accordance with both the type of work that the LHPs do and the CBPR programme that the current study was a part of, and also to get a deeper understanding of and gain insights into the experiences of the participants. The data collection for this study had to be modified according to the restrictions during the COVID-19 pandemic, as it had to be adapted to the situation. Thus, instead of the initially planned focus group discussions with the LHPs, individual interviews were conducted via Zoom. Discussions and reflections in focus groups might have been deeper given that the LHPs would have been able to reflect together with the other health promoters; however, the priority to obtain the LHPs’ views at the appropriate time outweighed the methological considerations.

During the focus group interviews with the citizens, the LHPs had to act as interpreters, as the participants mainly spoke Arabic. When using an interpreter to conduct interviews, possible threats to validity arise. Even if the benefits of using an interpreter are greater than the disadvantages, attention must be paid when transmitting messages between the two sets of people as the interpreter may misinterpret the messages. The data were analysed using inductive content analysis. Using this analytical approach made it possible to structure the results from the focus groups and the individual interviews and present them under relevant themes. As there is always a risk of subjectivity in data interpretation and texts can be interpreted in different ways, the authors engaged in continuous discussions during the analysis process until high intersubjectivity agreement was reached. The inclusion of quotations from the participants gives the opportunity for the readers to judge the trustworthiness of the study [33].

## Conclusions

To achieve sustainability in the future, community-based health promotion programmes must be able to manage change, be more flexible and maintain their work through difficult crises. The role of LHPs in the EU has mostly been related to non-communicable diseases, but the roles of brokers in the civil society are even more important in relation to communicable diseases, particularly when becomes a pandemic. The citizens felt that the reflective participatory dialogues and activities with the LHPs were crucial in the pandemic, in balancing between different information. In addition, the use of LHPs as brokers should also be considered by the PHA, as one of the key strategies to effectively achieve collective actions during a pandemic. To reduce epistemic injustice mere linear knowledge transfer may not suffice but rather dialogues, and oriented actions through co-learning in a local community context is recommended especially during a crisis situation as COVID-19.

## Data Availability

The data from the current study are not publicly available but can be requested from the corresponding author.
